# ZnO Nanostructures with Antibacterial Properties Prepared by a Green Electrochemical-Thermal Approach

**DOI:** 10.3390/nano10030473

**Published:** 2020-03-05

**Authors:** Maria Chiara Sportelli, Rosaria Anna Picca, Margherita Izzi, Gerardo Palazzo, Roberto Gristina, Massimo Innocenti, Luisa Torsi, Nicola Cioffi

**Affiliations:** 1 Chemistry Department, University of Bari “Aldo Moro”, via E. Orabona 4, 70126 Bari, Italy; maria.sportelli@uniba.it (M.C.S.); margherita.izzi@uniba.it (M.I.); gerardo.palazzo@uniba.it (G.P.); luisa.torsi@uniba.it (L.T.); nicola.cioffi@uniba.it (N.C.); 2 CSGI (Center for Colloid and Surface Science) c/o Dept. Chemistry, via Orabona 4, 70125 Bari, Italy; 3 Institute of Nanotechnology, National Research Council of Italy (CNR-NANOTEC), c/o Department of Chemistry, University of Bari “Aldo Moro”, via Orabona 4, 70126 Bari, Italy; roberto.gristina@cnr.it; 4 Chemistry Department, University of Florence, Via Lastruccia 3-13, 50019 Sesto Fiorentino (FI), Italy; m.innocenti@unifi.it

**Keywords:** zinc oxide, electrochemical synthesis, BAC, PDDA, nanorod, XPS, TEM, *B. subtilis*

## Abstract

Zinc oxide (ZnO) nanostructures are widely applied materials, and are also capable of antimicrobial action. They can be obtained by several methods, which include physical and chemical approaches. Considering the recent rise of green and low-cost synthetic routes for nanomaterial development, electrochemical techniques represent a valid alternative to biogenic synthesis. Following a hybrid electrochemical-thermal method modified by our group, here we report on the aqueous electrosynthesis of ZnO nanomaterials based on the use of alternative stabilizers. We tested both benzyl-hexadecyl-dimetylammonium chloride (BAC) and poly-diallyl-(dimethylammonium) chloride (PDDA). Transmission electron microscopy images showed the formation of rod-like and flower-like structures with a variable aspect-ratio. The combination of UV–Vis, FTIR and XPS spectroscopies allowed for the univocal assessment of the material composition as a function of different thermal treatments. In fact, the latter guaranteed the complete conversion of the as-prepared colloidal materials into stoichiometric ZnO species without excessive morphological modification. The antimicrobial efficacy of both materials was tested against *Bacillus subtilis* as a Gram-positive model microorganism.

## 1. Introduction

Semiconductor metal oxide nanostructures, and specifically ZnO ones, have received a great amount of interest in many areas due to their unique physical, chemical and optical properties [1]. Moreover, ZnO nanomaterials are well-known antimicrobial agents [2], which can be employed in several fields where pathogen spreading should be inhibited [3,4,5]. Besides the many physical [6,7,8] and chemical [9,10,11] methods for the production of such materials, scientific research has recently focused on green and simple electrochemical approaches [12,13]. These synthetic routes have the great advantage of being completely eco-friendly, using mainly aqueous solutions in the absence of reductants or other harmful chemicals [14]. Moreover, they can be easily scaled up for industrial purposes, and are cheap and easily reproducible [12,15]. Electrochemical approaches for the production of unsupported ZnO nanostructures (ZnONSs) are divided into three categories: electrochemical deposition under oxidative conditions (EDOC), electrolysis in alcohols, and electrolysis in the aqueous phase. EDOC [16] involves three stages: oxidation of a sacrificial anode, cathodic reduction of metal ions, and oxidation of metal nanoclusters by means of the oxygen introduced into the cell, assisted by stabilization by surfactants. Electrolysis in alcohols consists in the electrochemical degradation of a Zn electrode at high potentials in an anhydrous environment [17,18]. This process was recently updated by Dhayagude et al. [19] using tetra-butyl-ammonium bromide (TBAB) as a stabilizer in a water/ethanol electrochemical medium. Regarding the synthesis in the aqueous alkali phase, a hybrid electrochemical-thermal method was proposed in 2010, which used an aqueous solution of NaHCO_3_ to generate mixed carbonate and hydroxide Zn species, which were subsequently converted into ZnO by a calcination process at temperatures >300 °C [15,20]. In our group, we modified this method, in order to obtain a higher level of morphological control in the produced nanostructures. Both anionic [14,21] and cationic [22] stabilizers were dispersed in the electrochemical media and tested, and we demonstrated that we were able to produce various morphologies, ranging from spheroidal particles to rice-grain or rod-like structures.

Here, we propose a further upgrade of the aforementioned electrochemical process, which is run under mild galvanostatic conditions, using either benzyl-dimetylammonium chloride (BAC) or poly-diallyl-(dimethylammonium) chloride (PDDA) as a stabilizer. The first is a well-known disinfectant, and was used with the intent of producing nanostructures with enhanced antimicrobial properties [23,24]. The second one, a high charge density cationic polymer, is already used for the preparation of transistors and sensors, and as a coadjuvant in layer-by-layer deposition processes [25]. The gel-like products of the electrochemical step underwent a two-step thermal treatment: overnight desiccation at 120 °C and calcination at 450 °C for 1 h. Both the dried and the calcined samples, prepared with the two stabilizers, were characterized by transmission electron microscopy (TEM), and UV–Vis, infrared (FTIR) and *X*-ray photoelectron (XPS) spectroscopies. The critical role of temperature on the physicochemical properties of the final nanostructures was demonstrated. The antimicrobial activity of both the BAC- and PDDA-stabilized ZnONSs was demonstrated against a model Gram-positive microorganism, i.e., *B. subtilis*.

## 2. Materials and Methods

### 2.1. Materials

Zinc sheets (1 mm thick, 99.99+%) were purchased from Goodfellow Ltd and cut into 2 × 1 cm^2^ pieces. Sodium bicarbonate (NaHCO_3_, purum p.a., 99.0%), 2-propanol (IPA, Chromasolv^®^ Plus, for HPLC, 99.9%), hydrochloric acid (HCl, ACS reagent, 37%), benzyl-hexadecyl-dimetylammonium chloride (BAC, pure cationic surfactant), potassium bromide (KBr, FTIR grade, >99% trace metals basis), poly-diallyl-(dimethylammonium) chloride (PDDA, with an average molecular weight MW of 200,000–350,000, 20 wt.% in H_2_O), and Luria-Bertani (LB) broth (Miller, pH 6.8–7.2, 2.5% solution) were purchased from Merck-Sigma Aldrich (Milan, Italy). Aluminum oxide (Al_2_O_3_, purum p.a., 99.7%), for the mechanical polishing of zinc sheets, was obtained from Fluka Chemicals (Milan, Italy). Agar powder, meat extract, and peptone (from casein, pancreatic digest) were supplied by SIFIN Diagnostics Gmbh (Berlin, Germany). Milli-Q water was used in all experiments.

### 2.2. Synthesis of ZnONSs

A three-electrode setup was employed, using two zinc sheets as working (WE) and counter (CE) electrodes, and an Ag/AgCl (KCl sat.) as a reference electrode (RE). First, zinc electrodes were polished using sandpaper and afterward using alumina slurries with a different granulometry. Then, they underwent sonication, alternating between MilliQ water and IPA. Electrodes were finally activated in 1 M HCl for 30 s prior to use. The process yield (in terms of the mass of produced ZnONSs) was estimated by a differential weighting of the two zinc sheets before and after the process. The electrosynthesis was carried out in mild galvanostatic conditions, applying a current density of 10 mA/cm^2^ with a CH-1140b potentiostat-galvanostat (CH Instruments, Bee Cave, TX, USA). The process was performed under continuous stirring for 1 h, at room temperature. The electrolytic medium was composed of either 0.01 M BAC or 1 g/L PDDA, dissolved in 30 mM NaHCO_3_ aqueous solution. pH was monitored at the beginning and the end of the synthesis. Afterward, the colloidal dispersion was centrifuged at 6000 rpm for 30 min, and the resulting precipitate was dried overnight at 120 °C. The obtained powder underwent calcination at 450 °C in a tubular muffle furnace for 1 h.

### 2.3. Morphological and Spectroscopic Characterizations

TEM microscopy was performed with an FEI Tecnai 12 (Hillsboro, OR, USA) instrument (high tension: 120 kV; filament: LaB_6_), by dropping NP suspensions on a Formvar^®^-coated Cu grid (400 mesh, Agar Scientific, Stansted, UK).

UV–Vis spectroscopy was carried out with a Shimadzu UV-1601 double beam spectrometer, equipped with a silicon photodiode detector, from 200 to 700 nm, with 1-cm Quartz Suprasil^®^ (Hellma Analytics, Jena, DE) cuvettes.

Infrared spectra were recorded on a Perkin Elmer Spectrum-Two (Milan, IT), in the spectral range of 4000–400 cm^−1^, with a resolution of 1 cm^−1^, averaging 8 consecutive scans. The samples were analyzed as KBr pellets by grinding a proper quantity of the ZnO nanopowder in an agate mortar.

XPS surface analysis was performed on a PHI (Chanhassen, MN, USA) Versaprobe II spectrometer. A monochromatized Al-Kα source (1486.6 eV) was used. Dual-beam charge neutralization was constantly applied during the analysis. Large-area XPS was performed, operating with a sampling area of 200 × 1400 μm^2^. Samples were mounted onto the sample holder by means of double-sided tape. Survey scans (pass energy = 117.4 eV, step size = 1 eV) and high-resolution regions (pass energy = 58.7 eV, step size = 0.125 eV) relevant to C1s, O1s, N1s, Na1s, Cl2p, Zn2p, and ZnLMM were investigated. Detailed spectra processing was performed by CasaXPS^®^ (v. 2.3.18PR1.0) software. Binding Energy (BE) referred to the aliphatic component of C1s at 284.8 eV. For estimating the proper peak positions and assignments in both IR and XPS analyses, a commercial ZnO powder, obtained from Sigma Aldrich (Milan, IT), was used.

Aqueous ZnONS suspensions of 0.5 g/L were also characterized by ζ-potential measurements, using a Zetasizer-Nano ZS from Malvern Instruments (Rome, IT). The cell holder was maintained at 25 °C by a Peltier element; Laser-Doppler electrophoresis (LDE) exploited forward scattering at 17°. The LDE measurements were performed in a disposable capillary cell, and the ζ-potential was evaluated from the electrophoretic mobility according to the Smoluchowski approximation.

### 2.4. Antimicrobial Activity of ZnONSs

BAC- and PDDA-ZnONSs were suspended in Milli-Q water at a concentration of 0.15% w/v and deposited by drop casting on sterilized circular glass slides (⌀ 12 mm, Agar Scientific). The *B. subtilis* isolated strain (MTCC 441) was provided by the Institute of Bioscience and Biology of the University of Bari. Fifty milliliters of fresh LB broth was inoculated with 1 mL of cell suspension and incubated at 30 °C. The bacterial culture at the beginning of the exponential growth phase, at a concentration of 10^7^ CFU/mL (colony forming unit), was seeded (200 µL) on LB-agar Petri dishes. Then, circular glass slides covered with ZnONSs were put in contact with the seeded plates and incubated at 30 °C for 40 h. Experiments were performed in 5 replicates. The average values were used for calculation of the inhibition zone area. The minimum diameter of the inhibition zone was measured in mm.

A bare glass slide was used as a control sample.

## 3. Results and Discussion

### 3.1. Electrochemical Production of ZnONSs

This work follows the steps of the electrochemical-thermal hybrid method proposed by Chandrappa et al. [20], and improved by our group with the use of both cationic [22] and anionic [14,21] stabilizers. A colloidal suspension of hydroxides and zinc carbonates was produced in the presence of PDDA and BAC. As already shown [14,21,26], in fact, the final pH of the electrolytic medium is always highly basic (>9), and species such as Zn(OH)_2_, [Zn(OH)_3_]^−^ and [Zn(OH)_4_]^2-^ are thermodynamically stable in these conditions. The mixture is then converted into pure ZnO by thermal treatments. For each synthesis, the electrodes were weighed before and after the process, in order to calculate the mass variation and the experimental yield (Equation (1)).

(1) $$m_{experimental}=\;\Delta m\left(WE\right)-\;\Delta m\left(CE\right)$$

The theoretical mass was calculated according to Faraday’s law for a two-electron process, as expressed in Equation (2), where *I* is current intensity, Δ*t* is process duration, *M_Zn_* is Zn atomic mass, *Z* is the number of electrons involved in the process, and *F* is the Faraday constant:

(2) $$m_{theoretical}=\frac{M_{Zn}\left(\frac{g}{mol}\right)\;\times \Delta t\left(s\right)\times I\left(A\right)}{Z\left(\#e^{-}\right)\times F\frac{C}{mol}}$$

The obtained results, for both stabilizers, are listed in Table 1.

The synthesized products were first subjected to a desiccation treatment in an oven at 120 °C, which was carried out overnight, and a further thermal treatment at 450 °C for 1 h.

### 3.2. Morphological Characterization

**Figure 1:**
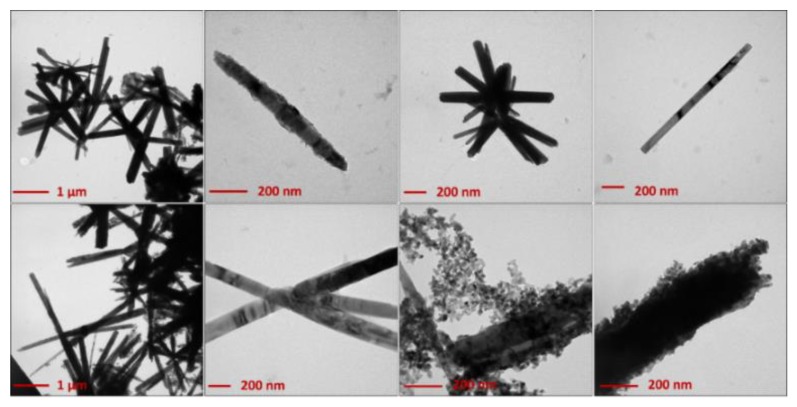
Transmission electron microscopy (TEM) images of electrosynthesized ZnO-based materials in the presence of benzyl-dimetylammonium chloride (BAC). Upper panels: ZnO dried at 120 °C overnight; lower panels: ZnO calcined at 450 °C for 1 h.

Morphological analyses on both dried (120 °C) and annealed (450 °C) ZnONSs exhibited the influence of both stabilizers and thermal treatments. Figure 1 shows the TEM micrographs obtained on the ZnO nanostructures that were prepared in the presence of BAC.

Rod-like and flower-like structures (with an average length of 1–2 µm) were obtained from samples dried at 120 °C. This morphology was preserved after calcination at 450 °C. However, a certain number of spheroidal nanoparticles (with an average diameter >5 nm) appeared after calcination, mainly at the wire tips. This phenomenon can be indicative of a possible mechanism of wire growth induced by calcination. In fact, the calcined samples showed slightly longer nanowires. The role of BAC in the growth mechanism of elongated rod-like structures may be tentatively explained by considering certain similarities between the effect of BAC and that exerted by cetyltrimethylammonium bromide (CTAB), which is an asymmetric quaternary ammonium salt, as well [27]. It has been reported in the literature that CTA^+^ ions, which have a structure of a charged tetrahedron with a long hydrophobic tail, can electrostatically interact with [Zn(OH)_4_]^2−^ (anion present in solution with a tetrahedral geometry, known to be the growth unit for ZnO), forming ion pairs [28,29,30]. In this way, the lateral growth is inhibited, whereas it is promoted along the *c*-axis ([0 0 0 1] direction), thus favoring the hexagonal (wurtzite) rod-like morphology [28]. Both CTAB and BAC promote the growth of ZnO crystallites in the form of wurtzite, the most thermodynamically stable ZnO crystalline form. Moreover, heating can favor assembly into flower-like shapes [28].

**Figure 2:**
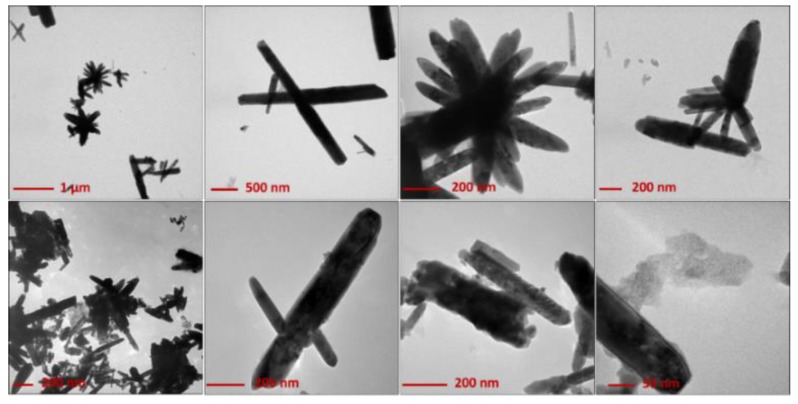
TEM images of electrosynthesized ZnO-based materials in the presence of poly-diallyl-(dimethylammonium) chloride (PDDA). Upper panels: ZnO dried at 120 °C overnight; lower panels: ZnO calcined at 450 °C for 1 h.

Analogously, Figure 2 reports images relevant to ZnO synthesized in the presence of PDDA.

The latter promoted the formation of flower-like microstructures, with an average wire length of 200–500 nm. This morphology was also preserved after calcination, although the thermal deterioration of the polymer tended to produce more aggregates. In the case of PDDA, a growth mechanism involving lamellar superimposition can be hypothesized, as shown by the growth lines running along wires’ length (Figure S1). Typically, the ZnO nanophases obtained by the proposed method show a crystalline, wurtzite structure, with a significant number of defects, as assessed by selected-area electron diffraction (SAED) analysis (Figure S2). Interestingly, other results indicate that calcination is not essential to obtain ZnO when using PDDA (vide infra).

### 3.3. FTIR and UV–Vis Characterizations

**Figure 3:**
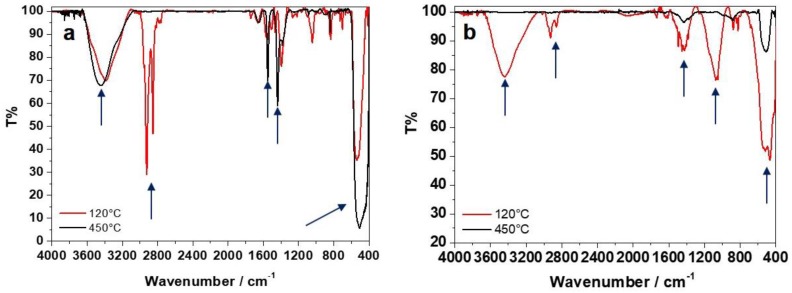
The FTIR spectra of dried (120 °C) and calcined (450 °C) samples: (**a**) ZnO-BAC; (**b**) ZnO-PDDA. The main IR modes are highlighted by arrows.

The FTIR spectra of all samples are presented in Figure 3.

In the case of BAC (Figure 3a), IR data indicate that ZnO is already present in the 120 °C-dried samples, as highlighted by the characteristic IR band at 440–500 cm^−1^ attributed to the Zn-O stretching [22]; the conversion of Zn(II) species into stoichiometric ZnO is complete with calcination at 450 °C. The region between 1300 and 1600 cm^−1^ is typically associated with the presence of hydrozincite-like species (Zn_x_(CO_3_)_y_(OH)_z_) [31] and lowers after calcination. Furthermore, the increase in the treatment temperature also led to the disappearance of C-H stretching (3000–2800 cm^−1^) signals [32]; on the contrary, hydroxyl (3500–3400 cm^−1^) and styrene/carbonyl bands (1700–1500 cm^−1^) slightly increase upon calcination, as the effect of a possible BAC degradation with temperature, forming burnt moieties.

Regarding PDDA (Figure 3b), the IR spectrum of the dried sample presented some characteristic bands of PDDA, not degraded by the thermal treatment. The absence of the two main absorptions related to the symmetric and asymmetric stretching modes (with the characteristic double-pointed shape) of the carbonates at 1389–1500 cm^−1^ [33] and of the other characteristic signals at 1047 cm^−1^, 837 cm^−1^, and 710 cm^−1^, combined with the presence of the 3400-cm^−1^ band relative to O-H stretching (H_2_O and hydroxides), indicates that Zn^2+^ is mainly present as hydroxide and not as hydrozincite. On the other hand, the IR spectrum of the calcined sample showed a stronger signal due to Zn-O stretching, whereas the hydroxide band decreased. Moreover, signals related to the polymeric stabilizer were no longer visible after calcination at 450 °C.

**Figure 4:**
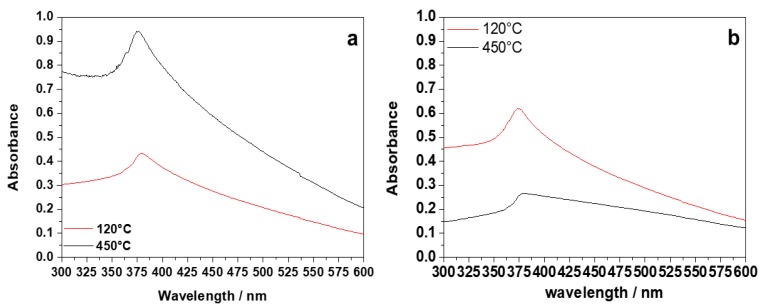
UV–Vis spectra of dried (120 °C) and calcined (450 °C) samples: (**a**) ZnO-BAC; (**b**) ZnO-PDDA.

The UV–Vis spectra of all of the samples are presented in Figure 4. In the case of BAC (Figure 4a), the UV–Vis spectrum of the dried sample exhibited an absorption peak ascribable to ZnO (350–400 nm). The calcination process induced an increase in the peak intensity, due to pure ZnO and to a stronger absorption. For PDDA (Figure 4b), the band ascribable to ZnO showed a slight red shift from 374 nm to 382 nm with the increase in the treatment temperature, which could be related to a nanostructure size increase (a phenomenon generally reported in the literature) [14]. Broad absorption at higher wavelengths is attributed in the literature to crystal defects, especially oxygen vacancies in the sample [34].

### 3.4. XPS Characterization

**Figure 5:**
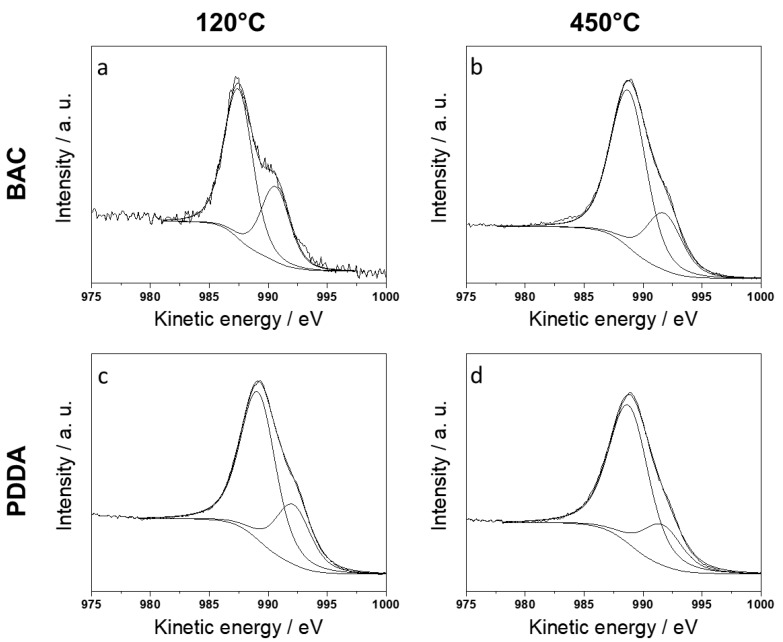
ZnL_3_M_4,5_M_4,5_ XP spectra of dried (120 °C) and calcined (450 °C) samples. ZnO-BAC (**a**,**b**); ZnO-PDDA (**c**,**d**).

XPS was useful in the univocal determination of zinc chemical speciation. Zn(II) species can be discriminated by means of the ZnL_3_M_45_M_45_ Auger signal (expressed in kinetic energy, KE) and of the modified Auger parameter α′. In fact, the Zn2p_3/2_ photoelectronic signal is uninformative as the chemical shift on this signal is small, compared to that on the corresponding Auger maximum. In fact, typical values for Zn2p_3/2_ ranging between 1021 and 1023 eV are indifferently reported for zinc metal or Zn(II) [35]. All the analyzed samples presented Zn2p_3/2_ positions in this range (Table 2). This means that the correct zinc speciation, based only on the main photoelectronic peak, is unreliable [36,37,38]. Considering that the typical value of α′ reported for ZnO is equal to 2010.2 ± 0.3 eV [14,22,26,35,39,40], the Zn Auger signal was acquired and analyzed to assess the presence of zinc oxide in the investigated samples (Figure 5). The main component of ZnL_3_M_4,5_M_4,5_ was used for the further calculation of α′ and it is mainly ascribed to the ^1^G final state transition, which is known to be the most probable [37,41]. In the case of BAC, XPS data revealed that the increase in the calcination temperature led to the increment of α′ (Table 2), reaching values compatible with the presence of ZnO on the surface of the nanostructures. Dried samples presented a low value of α′ (compatible with zinc hydroxides) [39], whereas it was around 2010.0 eV for 450 °C-calcined samples. These results are in agreement with the IR data. The XPS data on ZnO-BAC samples treated at 450 °C did not show any signal ascribable to nitrogen. This is indicative of the non-persistence of the cationic stabilizer on ZnONSs after calcination, and is in agreement with the IR results.

In a similar way, the XPS results on the PDDA-based samples are corroborated by the IR findings. The modified Auger parameter for zinc was compatible with the presence of ZnO, already in dried samples at 120 °C. The obtainment of pure ZnO in milder conditions with respect to the current state-of-the-art [14,21] makes ZnO-PDDA a highly appealing material. It is worth pointing out that, for the ZnO-PDDA treated at 120 °C, the N1s signal (Figure S3) that resulted is made up of two components: the first at a binding energy of 399.2 ± 0.2 eV, and the second at 402.2 ± 0.2 eV. They were associated with the presence of free amine groups −N(CH_2_)/−NH_2_, and protonated groups (mainly −N(CH_3_)_3_^+^/−NH_3_^+^) [35]. This evidence can be indicative that the quaternary nitrogen of the PDDA has partly been preserved and that the cationic character of the polymer has not been completely lost.

### 3.5. ζ-Potential Measurements

**Figure 6:**
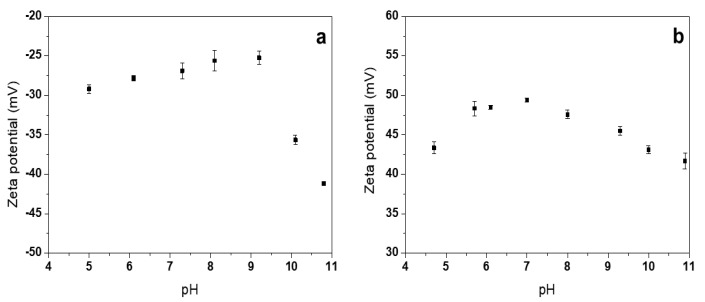
ζ-potential measurements as a function of pH: (**a**) calcined (450 °C) ZnO-BAC nanostructures; (**b**) dried (120 °C) ZnO-PDDA nanostructures.

ζ-potential measurements (Figure 6) were performed on the most interesting samples in terms of chemical composition, morphology, and ease of preparation. From the characterizations reported above, it appears clear that ZnONSs prepared with BAC benefit from a calcination step when stoichiometric ZnO phases are desired. During this step, the stabilizing agent degrades and forms burnt carbonaceous moieties on nanostructures. This could be the reason why the calcined ZnONSs @BAC did not show any isoelectric point and had a highly negative ζ-potential in the pH range investigated here (Figure 6a).

In the case of ZnO-PDDA, a mild drying process at 120 °C is enough to obtain ZnO. Moreover, as demonstrated by XPS, the polyelectrolyte is retained on the ZnO surface after the thermal treatment at 120 °C. The persistence of the cationic NP stabilizer is consistent with the presence of a positive and almost constant ζ-potential, with a pH ranging from 5 to 11 (Figure 6b).

### 3.6. Agar Disk Diffusion Tests

**Figure 7:**
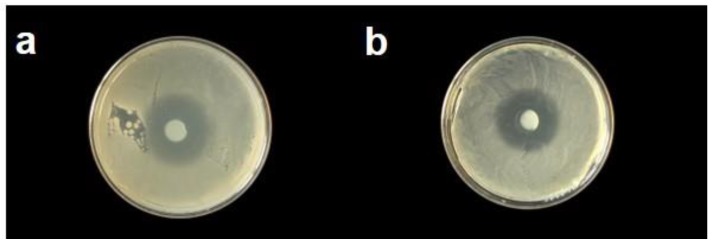
Evaluation of antibacterial action by an Agar diffusion test: (**a**) calcined (450 °C) ZnO-BAC nanostructures; (**b**) dried (120 °C) ZnO-PDDA nanostructures.

Antibacterial activity studies were carried out on both calcined (450 °C) ZnO-BAC nanostructures and dried (120 °C) ZnO-PDDA nanostructures. They showed consistent inhibition (>10 mm) [42] and no significant variation amongst stabilizers (Figure 7). In fact, the average inhibition diameters measured on five replicates resulted equal to 10.8 ± 0.6 mm for ZnO-BAC and 10.3 ± 1.2 mm for ZnO-PDDA. Slight differences in NP morphology and opposite ζ-potential values did not seem to have any influence on the NP antimicrobial behavior against a Gram-positive bacterial strain. No bacterial growth inhibition was found on the control samples.

## 4. Conclusions

In summary, we proposed a green electrochemical strategy for the synthesis of ZnONSs in aqueous alkaline media with low-cost, non-toxic chemicals under mild conditions. ZnO rod- and flower-like structures were successfully synthesized by employing two different stabilizers, namely, BAC and PDDA. While for BAC a thermal treatment at temperatures ≥450 °C was necessary for the complete conversion of the as-prepared gel-like material into ZnO, PDDA allowed for the preparation of pure ZnONSs without the need for severe thermal treatment. In fact, both FTIR and XPS measurements confirmed the presence of stoichiometric ZnO after a simple drying step at 120 °C. In all cases, TEM analysis revealed the presence of elongated (or rod-like) structures, generally assembled into more complex and ordered aggregates after calcination at 450 °C. Both BAC- and PDDA-containing materials exhibited a consistent antimicrobial efficacy against *B. subtilis*, as demonstrated by agar diffusion tests. The approach presented here can be considered as an improvement of the current methodologies to produce elongated ZnO nanomaterials in an aqueous solution, employing cationic capping agents, thanks to higher yields and milder preparation conditions [22]. Application of these ZnO nanostructures in transistor devices (PDDA-capped) and for cultural heritage preservation (BAC-capped) is envisaged, and work is scheduled for the future in this field.

## Figures and Tables

**Table 1:** The process efficiency for the preparation of nanocolloids, expressed in terms of nanodispersed Zn masses, and % yields.

Stabilizer	*Δm_WE_ (mg)*	*Δm_CE_ (mg)*	*m_experimental_ (mg)*	*m_theoretical_ (mg)*	% Yield
BAC	19 ± 2	0 ± 2	19 ± 4	24.4	79
PDDA	17 ± 2	0 ± 2	17 ± 4	24.4	70

**Table 2:** Zn2p, ZnL_3_M_4,5_M_4,5_ positions, and corresponding modified Auger parameter (α′) for all of the samples, as a function of the stabilizer and of the thermal treatment.

	Thermal Treatment	Zn2p_3/2_ BE (eV)	ZnL_3_M_4,5_M_4,5_ KE (eV)	α′ (eV)
**BAC**	120 °C	1022.0 ± 0.2	987.4 ± 0.2	2009.4 ± 0.3
	450 °C	1021.3 ± 0.2	988.8 ± 0.2	2010.1 ± 0.3
**PDDA**	120 °C	1021.1 ± 0.2	987.9 ± 0.2	2010.0 ± 0.3
	450 °C	1021.2 ± 0.2	988.8 ± 0.2	2010.0 ± 0.3

## Supplementary Materials

The following are available online at https://www.mdpi.com/2079-4991/10/3/473/s1, Figure S1: TEM image of dried (120 °C) ZnO-PDDA sample. Figure S2: Selected area diffraction (SAED) image obtained on ZnONSs. Figure S3: N1s XP spectrum of ZnO-PDDA dried at 120 °C.
