# Supplementary material for: ZnO Nanostructures with Antibacterial Properties Prepared by a Green Electrochemical-Thermal Approach
Source: Nanomaterials (Basel). 2020 Mar 5;10(3):473. doi: 10.3390/nano10030473 (PMC7153254; doi:10.3390/nano10030473)
Supplement: Supplementary file 1 [file nanomaterials-10-00473-s001.pdf]

Electronic supplementary material

ZnO nanostructures with antibacterial properties prepared by a green electrochemical-thermal approach

Maria Chiara Sportelli[1, 2], Rosaria Anna Picca[1, 2*], Margherita Izzi[1], Gerardo Palazzo[1, 2], Roberto Gristina[3], Massimo Innocenti[4], Luisa Torsi[1, 2], and Nicola Cioffi[1, 2]

[1] Chemistry Department, University of Bari "Aldo Moro", via E. Orabona 4 – 70126 Bari, Italy. maria.sportelli@uniba.it; rosaria.picca@uniba.it; margherita.izzi@uniba.it; gerardo.palazzo@uniba.it; luisa.torsi@uniba.it; nicola.cioffi@uniba.it.

[2] CSGI (Center for Colloid and Surface Science) c/o Dept. Chemistry, via Orabona 4, 70125 Bari, Italy.

[3] Institute of Nanotechnology, National Research Council of Italy (CNR-NANOTEC), c/o Department of Chemistry, University of Bari "Aldo Moro" via Orabona 4, Bari, 70126, Italy. roberto.gristina@cnr.it

[4] Chemistry Department, University of Florence, Via Lastruccia 3-13 – 50019 Sesto Fiorentino (FI), Italy. m.innocenti@unifi.it

* Correspondence: rosaria.picca@uniba.it.

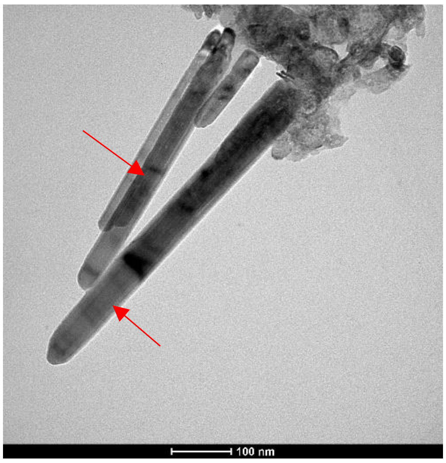

Figure S1: TEM micrograph of dried (120 °C) ZnO-PDDA sample. Wires growth lines are highlighted by arrows.

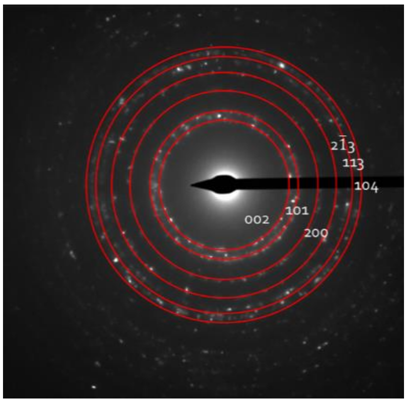

Figure S2: Selected area diffraction (SAED) image obtained on ZnO NSs.

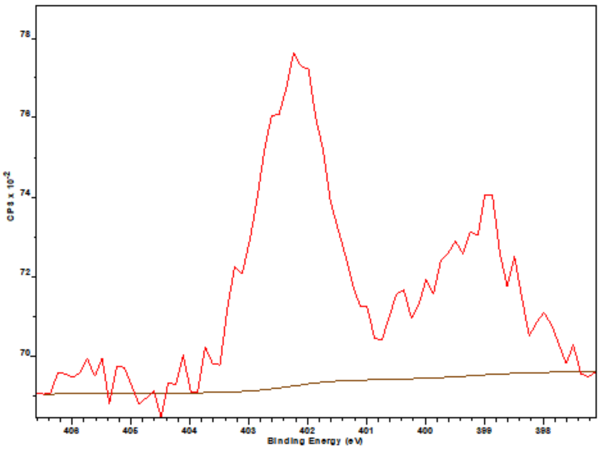

Figure S3: N1s XP spectra of dried (120 °C) ZnO-PDDA sample.
